# Could remifentanil reduce duration of mechanical ventilation in comparison with other opioids for mechanically ventilated patients? A systematic review and meta-analysis

**DOI:** 10.1186/s13054-017-1789-8

**Published:** 2017-08-03

**Authors:** Yibing Zhu, Yinhua Wang, Bin Du, Xiuming Xi

**Affiliations:** 1 Department of Critical Care Medicine, Fuxing Hospital, Capital Medical University, 20A FuXing Men Wai Da Jie, Xicheng District, Beijing, 100038 China; 2 Medical ICU, Peking Union Medical College Hospital, Peking Union Medical College and Chinese Academy of Medical Sciences, 1 Shuai Fu Yuan, Beijing, 100730 China; 3 Department of Critical Care Medicine, North China University of Science and Technology Affiliated Hospital, 73 Jianshe Road, Tangshan, 063000 China

**Keywords:** Analgesia, Remifentanil, Mechanical ventilation, Meta-analysis, Systematic review, Critical care

## Abstract

**Background:**

Sedation and analgesia are commonly required to relieve anxiety and pain in mechanically ventilated patients. Fentanyl and morphine are the most frequently used opioids. Remifentanil is a selective μ-opioid receptor that is metabolized by unspecific esterases and eliminated independently of liver or renal function. Remifentanil has a rapid onset and offset and a short context-sensitive half-life regardless of the duration of infusion, which may lead to reductions in weaning and extubation. We aimed to compare the efficacy and safety of remifentanil to that of other opioids in mechanically ventilated patients.

**Methods:**

We conducted a search to identify relevant randomized controlled studies (RCTs) in the PubMed, Embase, Cochrane Library and SinoMed databases that had been published up to 31 December 2016. The results were analysed using weighted mean differences (WMDs) and 95% confidence intervals (CIs).

**Results:**

Twenty-three RCTs with 1905 patients were included. Remifentanil was associated with reductions in the duration of mechanical ventilation (mean difference -1.46; 95% CI -2.44 to -0.49), time to extubation after sedation cessation (mean difference -1.02; 95% CI -1.59 to -0.46), and ICU-LOS (mean difference -0.10; 95% CI -0.16 to -0.03). No significant differences were identified in hospital-LOS (mean difference -0.05; 95% CI -0.25 to 0.15), costs (mean difference -709.71; 95% CI -1590.98 to 171.55; I^2^ 88%), mortality (mean difference -0.64; 95% CI -1.33 to 0.06; I^2^ 87%) or agitation (mean difference -0.71; 95% CI -1.80 to 0.37; I^2^ 93%).

**Conclusions:**

Remifentanil seems to be associated with reductions in the duration of mechanical ventilation, time to extubation after cessation of sedation, and ICU-LOS. No significant differences were identified between remifentanil and other opioids in terms of hospital-LOS, costs, mortality or agitation.

**Electronic supplementary material:**

The online version of this article (doi:10.1186/s13054-017-1789-8) contains supplementary material, which is available to authorized users.

## Background

Pain and anxiety are among the worst experiences for the critically ill, especially those on mechanical ventilation. Mechanically ventilated patients generally require a combination of analgesia and sedation to improve compliance with mechanical ventilation, adaption to endotracheal tubes, and pain relief [1]. Propofol and midazolam have been identified as the hypnotic drugs most commonly used for sedation in the intensive care unit (ICU), and among sedatives, shorter elimination half-lives are associated with shorter awakening times, resulting in reductions in time to weaning and time to extubation [2]. Opioids are commonly used in the ICU for analgesia, and the accumulation of opioid agents may cause respiratory depression, thus leading to prolongation of time to weaning and extubation.

Studies have found the most frequently used opioids to be fentanyl (30–35%), morphine (15–33%), and sufentanil (25–40%), the specific proportions of which differ in the results of different surveys, with remifentanil being less commonly used (10%) [3, 4]. However, the use of opioids may not be ideal in mechanically ventilated critically ill patients. The renal metabolism of morphine results in accumulation of morphine-6-glucuronide in patients with renal impairment. In addition, common adverse effects of morphine include histamine release, pruritus, and constipation [5]. Fentanyl, alfentanil and sufentanil undergo hepatic metabolism, and continuous infusion results in accumulation and prolongation of effect [5]. Those pharmacodynamic and pharmacokinetic profiles have disadvantages in terms of rapid weaning and extubation.

Similar to fentanyl, alfentanil, and sufentanil, remifentanil is a potent, selective 4-anilidopiperidine μ-opioid analgesic. However, unlike fentanyl and other opioids, remifentanil is completely metabolized by unspecific esterases in a manner that is independent of liver or renal function [5, 6]. Since unspecific esterases have been found to be widely distributed in living human cells, there are no ICU disease states or types of organ failure that would lead to reduced breakdown of remifentanil. The major breakdown product of remifentanil is remifentanil acid (RA), which has a potency of only 1/300 to 1/4600 that of remifentanil, has poor brain penetration and is unlikely to cause respiratory failure [6, 7]. Remifentanil has a highly predictable onset and offset effect, a terminal half-life of approximately 10 to 20 minutes, and a context-sensitive half-life of 3 to 4 minutes [6, 8]. The unique pharmacokinetics of remifentanil may lead to reductions in time to weaning and extubation and, accordingly, may be associated with reductions in mechanical ventilation time, length of ICU stay (ICU-LOS), and costs.

Several randomized controlled studies (RCTs) and cohort studies have been conducted to compare the efficacy and safety of remifentanil with those of other opioids. Mechanical ventilation time and extubation time are commonly evaluated indicators. Taking Dahaba et al.’s study [1] as an example, 40 mechanically ventilated patients who were assigned an analgesia protocol involving the administration of either remifentanil or morphine; these analgesics were initiated at the minimum dose and titrated up to an optimal level of sedation, with midazolam serving as a rescue treatment. The results of this study showed that remifentanil was associated with significant reductions in mean duration of mechanical ventilation and extubation time. The same outcome measures have been assessed in several RCTs). The results of a meta-analysis [9] showed that remifentanil was associated with reduced time to extubation after sedation cessation, but no significant difference was identified between remifentanil and other opioids in terms of mechanical ventilation duration. However, another meta-analysis [10] showed that remifentanil was associated with a significantly reduction in the duration of mechanical ventilation. The results of a cost-consequence analysis performed by Al et al. [11] suggested that remifentanil was associated with significantly decreased ICU costs, whereas the results of Engoren et al.’s study [12] showed that higher opioid and anaesthetic costs but lower hospital costs were identified in remifentanil group relative to the fentanyl group. The results of these economic analyses were complex. On the one hand, reductions in ventilation time, ICU-LOS and length of stay in hospital (hospital-LOS) were associated with a reduction in overall cost, but shorter-acting anaesthetics were more expensive. On the other hand, morphine withdrawal-associated immunosuppression and remifentanil discontinuation were identified as independent risk factors for ICU-acquired infections, and excessive analgesia was associated with nosocomial pneumonia delirium and psychological disorders, which increases the complexity of the evaluation of remifentanil [13]. Several studies have been conducted since the meta-analysis was performed in 2009 [10], which showed that remifentanil was associated with reduced time to extubation after sedation cessation and indicated the presence of no significant differences between remifentanil and other opioids in terms of mortality, duration of mechanical ventilation, ICU-LOS, and risk of agitation. Therefore, we conducted this study with the intention of updating these data and re-evaluating the efficacy and safety of remifentanil in mechanically ventilated patients relative to the safety and efficacy of other opioids.

## Methods

### Search strategy and selection criteria

Four electronic databases were searched (PubMed, Embase, Cochrane Library and SinoMed)) to identify studies published from 2001 until December 2016. A search strategy was developed for PubMed (Additional file 1: Appendix 1) and the other databases. Our research was limited to RCTs, and no language restriction was applied. The reference lists of relevant articles were also reviewed. We contacted the authors of the studies if additional data were required for the predefined outcomes. Non-English language articles were translated before further analysis.

Studies were included if they met the following criteria: (1) the study population consisted of adults (mean age ≥18 years old) undergoing mechanical ventilation; (2) the study design was an RCT; (3) remifentanil or a remifentanil/sedative combination was used for analgesia and sedation; and (4) the outcomes included at least one of the following measures: duration of mechanical ventilation, time to extubation after cessation of sedation, ICU-LOS, hospital-LOS, costs, proportion of patients with agitation, delirium, nausea/vomiting, or mortality.

Studies in which remifentanil was not compared with another opioid or another opioid/sedative combination were excluded. Publications available only in abstract form or as meeting reports were excluded.

### Data extraction and quality assessment

Two reviewers (ZYB and WYH) independently extracted data from the published sources using a predesigned data extraction form. The following data were abstracted from each included study: the study ID, journal, year of publication, country, setting, centre, mean age, proportion of male subjects, disease severity, disease type, proportion of post-surgical patients, sample size, comparator, inclusion criteria, exclusion criteria, intervention and outcomes. Two reviewers independently rated the quality of the RCTs using the Modified Jadad scores [14], which are determined using a checklist designed to measure the quality of RCT reporting. The following elements are evaluated when calculating a Modified Jadad score: randomization (0–2), concealment of allocation (0–2), double blinding (0–2), and withdrawals and dropouts (0–1). The trials were rated based on what they reported, and the results of the quality assessment are described in the table describing the characteristics of included studies (Table 1).

**Table 1:** Characteristics of included studies

Study ID	Journal	Year	Participants	Setting	Post-surgical patients	Interventions	Sedative	Aim	Outcomes
Al et al. [11]	*Critical Care*	2010	205 mechanically ventilated medical and surgical patients; mean age 65; mean SAPS II 45	ICU	37–44%	Remifentanil group: remifentanil 0.4–45ug/kg/h, propofol as rescue treatment once remifentanil infusion > 12 ug/kg/h was needed; control group: analgesia was achieved by either morphine 1–0 mg/h or fentanyl 25–100 mg/h, either propofol 0.5–4 mg/kg/h or midazolam 0.01–0.2 mg/kg/h or lorazepam 0.01–0.1 mg/kg/h for sedation	Remifentanil group: propofol 65%; control group: propofol 46%, midazolam 81%, lorazepam 7%	SAS 3–4	Time to extubation after cessation of sedation, duration of mechanical ventilation, ICU-LOS, costs
Rozendaal et al. [16]	*Intensive Critical Medicine*	2009	205 mechanically ventilated medical and surgical patients; mean age 65; mean SAPS II 45	ICU	37–44%	Remifentanil group: remifentanil 0.4–45 ug/kg/h, propofol as rescue treatment once remifentanil infusion > 12 ug/kg/h was needed; control group: analgesia was achieved by either morphine 1–10 mg/h or fentanyl 25–-100 mg/h, either propofol 0.5–4 mg/kg/h or midazolam 0.01–0.2 mg/kg/h or lorazepam 0.01–0.1 mg/kg/h for sedation	Remifentanil group: propofol 65%; control group: propofol 46%, midazolam 81%, lorazepam 7%	SAS 3–4	Time to extubation after cessation of sedation, ICU-LOS, duration of mechanical ventilation
Spies et al. [13]	*Intensive Critical Medicine*	2011	65 adult medical and surgical patients requiring mechanical ventilation for more than 24 h; mean age 63; mean APACHE II 25; mean SOPA 9	ICU	92–97%	Remifentanil group: remifentanil 0.1–0.4 ug/kg/min; fentanyl group: fentanyl 0.02–0.08 ug/kg/min; the study protocol did not allow any bolus application of either fentanyl or remifentanil	Remifentanil group:propofol, midazolam, lorazepam; fentanyl group: propofol, midazolam, lorazepam, proportion not mentioned	VAS ≤ 3 and/or BPS ≤ 6	Duration of mechanical ventilation, ICU-LOS, hospital-LOS, delirium, reflux/vomiting
Lui et al. [20]	*Zhonghua Wei Zhong Bing Ji Jiu Yi Xue*	2013	60 patients with mechanical ventilation for over 24 h after tumor operation; mean age 64; mean APACHE II 20	ICU	100%	Remifentanil group: remifentanil 0.05 ug/kg/h, titrated up with increment of 0.025 ug/kg/min; fentanyl group: fentanyl 0.5 ug/kg/h, titrated up with increment of 0.25 ug/kg/h; propofol as rescue treatment for remifentanil group once remifentanil infusion > 0.1 ug/kg/h and for fentanyl group once fentanyl infusion > 1 ug/kg/h	Remifentanil group: propofol 26%;fentanyl group: propofol 63%	Ramsay 2–3	Duration of mechanical ventilation, ICU-LOS, costs
Khanykin et al. [36]	*The Heart Surgery Forum*	2013	71 mechanically ventilated postoperative patients; mean age 64	ICU	100%	Remifentanil group: remifentanil 0.2–0.5 ug/kg/min as required after termination of bypass, remifentanil 0.1–0.2 ug/kg/min for postoperative pain; low-dose fentanyl group: fentanyl 3–4ug/kg as required after termination of bypass, morphine 2.5–5 mg for postoperative pain	None	NR	ICU-LOS, hospital LOS, extubation time
Bhavsar et al. [21]	*Anesthesia*	2016	60 mechanically ventilated postoperative patients; mean age 68	ICU	100%	Remifentanil group: remifentanil 0.4–0.6 ug/kg/min during surgery, remifentanil 0.1ug/kg/min after surgery; sufentanil group: sufentanil 1–2ug/kg within 1–2 minutes, total dose of sufentanil 3–3.5ug/kg before cardiopulmonary bypass	None	VAS 3–4	Duration of mechanical ventilation, ICU-LOS, hospital-LOS
Engoren et al. [12]	*Anesthesia & Analgesia*	2001	90 adult patients undergoing cardiac surgery; mean age 60	ICU	100%	Remifentanil group: remifentanil infusion at 0.5–1 ug/kg/min,then maintained 0.05–1 ug/kg/min; fentanyl group: fentanyl 7–10 ug/kg for the induction and additional doses of 1–2 ug/kg as needed for intense stimulus; sufentanil group: sufentanil 1–4 ug/kg for the induction and 0.1–0.3 ug/kg as needed for intense stimulus	None	NR	Duration of mechanical ventilation, ICU-LOS, hospital LOS, costs
Muellejans et al. [22]	*Critical Care*	2006	80 adult patients undergoing elective cardiac surgery; mean age 66; mean SAPS II 33	ICU	100%	Remifentanil group: remifentanil 6–60 ug/kg/h; fentanyl/midazolam group: fentanyl 1–2 ug/kg/h, midazolam 0.02–0.04 mg/kg/h; propofol as rescue treatment for remifentanil group and midazolam ± fentanil for fentanyl/midazolam group	None	VAS < 4	Delirium, time to extubation after cessation of sedation, duration of mechanical ventilation, ICU-LOS, costs
Muellejans et al. [23]	*Critical Care*	2004	152 mechanically ventilated medical or surgical patients; mean age 60; mean SAPS II 28	ICU	92–95%	Remifentanil group: remifentanil 9–12 ug/kg/h fentanyl group: fentanyl 1–2 ug/kg/h; propofol as rescue treatment for both groups	Remifentanil group: propofol 35%; fentanyl group: propofol: 40%	SAS 4	Proportion required rescue sedation, nausea/vomiting, time to extubation after cessation of sedation
Karabinis et al. [24]	*Critical Care*	2004	161 mechanical ventilated elective or emergency neurosurgical patients; mean age 47	neuro-ICU	25–49%	Remifentanil group: remifentanil 9-18 ug/kg/h; fentanyl group: fentanyl 0.1–7.9 ug/kg/min; morphine group: 0-–6.8 mg/kg/min; propofol as rescue treatment for remifentanil group and all patients changed to midazolam infusion on day 3	Remifentanil group: propofol 90%, midazolam 36%; fentanyl group: propofol 100%, midazolam 30%; morphine group: propofol 93%,midazolam 30%	SAS < 4	Mortality, time to extubation after cessation of sedation, duration of mechanical ventilation
Baillard et al. [25]	*Annales Françaises d'Anesthésie et de Réanimation*	2005	41 mechanically ventilated medical and trauma patients; mean age	ICU	29%	Remifentanil group: remifentanil 10 ug/kg/h and titrated; sufentanil group: sufentanil 0.125 ug/kg/h and titrated; current midazolam for both groups at 0.1 mg/kg/h	Both of remifentanil and sufentanil group: midazolam 100%	Ramsay 4	Mortality, time to extubation after cessation of sedation, ICU-LOS
Dahaba et al. [1]	*Anesthesiology*	2004	40 mechanically ventilated patients after orthopedic or general surgery; mean age 58; mean SAPS II 23	ICU	100%	Remifentanil group: remifentanil 9–12 ug/kg/h morphine group: morphine 0.04–0.06 mg/kg/h; routine current midazolam 0.03 mg/kg/h for both groups as rescue treatment	Remifentanil group: midazolam 30%; morphine group: 45%	SAS 4	Proportion of patients with agitation, nausea/vomiting, mortality, time to extubation after cessation of sedation, duration of mechanical ventilation, ICU-LOS
Breen et al. [26]	*Critical Care*	2005	105 mechanically ventilated 3–10days) patients; mean age 54; mean SAPS II 43	ICU	8–13%	Remifentanil group: remifentanil 6–18 ug/kg/h; fentanil or morphine group: standard clinical protocol for the unit; midazolam 2 mg bolus as rescue treatment for both groups	Remifentanil group: midazolam 74%; fentanyl group: midazolam 62%; morphine group: midazolam 23%	SAS 3–4	Nausea/vomiting, mortality, time to extubation after cessation of sedation; duration of mechanical ventilation, ICU-LOS
Belhadj Amor et al. [27]	*Annales Françaises d'Anesthésie et de Réanimation*	2007	19 mechanically ventilated patients with renal impairment(creatinine clearance < 50 ml/min); mean age 60; mean APACHE II 37	ICU	0	Remifentanil group: remifentanil 6 ug/kg/h, titrated up by increment of 100 ug/h; fentanyl group: fentanyl 1.5 ug/kg/h, titrated up with increment of 25 ug/h; routine concurrent midazolam infusion for both groups at 0.1 mg/kg/h	Remifentanil group: midazolam 78%; fentanyl group: midazolam 95%;	Ramsay 3-4	Proportion of patients with agitation, time to extubation after cessation of sedation, ICU-LOS
Carrer et al. [28]	*Minerva Anestesiologica*	2007	100 mechanically ventilated postsurgical patients; mean age 69; mean SAPS II 26	ICU	100%	Remifentanil group: remifentanil 6 ug/kg/h and titrated; morphine group: morphine 0.03–0.04 mg/kg/h and titrated; concurrent morphine for both groups at 0.01 mg/kg/h, diazepam 0.1 mg/kg as rescue treatment	Remifentanil group: diazepam 28%; morphine group: diazepam 60%	Ramsay 3	Proportion of patients achieving optimal level of sedation without rescue therapy, nausea/vomiting, duration of mechanical ventilation, ICU-LOS
Gerlach et al. [29]	*Journal of Cardiothoracic & Vascular Anesthesia*	2002	26 mechanically ventilated postsurgical patients; mean age 64	ICU	100%	Remifentanil group: remifentanil 0.15–0.3 ug/kg/min; sufentanil group: sufentanil 0.5–1 ug/kg/h	Both of remifentanil and sufentanil groups: propofol, proportion not mentioned	Self-reported no pain	Duration of mechanical ventilation, nausea/vomiting, time to extubation after cessation of sedation
Guggenherger et al. [30]	*European Journal of Anaesthesiology*	2006	59 mechanically ventilated postsurgical patients; mean age 67	ICU	100%	Remifentanil group: remifentanil 0.5–1 ug/kg/min; sufentanil group: 30–40 ng/kg/min	Both of remifentanil and sufentanil groups: propofol, proportion not mentioned	VAS < 4	Duration of mechanical ventilation, hospital-LOS, time to extubation after cessation of sedation, ICU-LOS
Knapik et al. [31]	*Medical Science Monitor*	2006	40 mechanically ventilated postsurgical patients; mean age 56	ICU	100%	Remifentanil group: remifentanil 0.25–0.5 ug/kg/min; fentanyl group: fentanyl 2.5 ug/kg/h	None		Duration of mechanical ventilation, hospital-LOS
Maddali et al. [32]	*Journal of Clinical Anesthesia*	2006	180 mechanically ventilated postsurgical patients; mean age 55	ICU	100%	Remifentanil group: remifentanil 1 ug/kg/min; fentanyl group: 0.025–0.15 ug/kg/min; diclofenac group: propofol 2–5 mg/kg/h	Both of remifentanil and fentanyl groups: propofol 100%	VAS < 4	Duration of mechanical ventilation, time to extubation after cessation of sedation, ICU-LOS
Myles et al. [19]	*Anesthesia and Analgesia*	2002	87 mechanically ventilated postsurgical patients; mean age 62	ICU	100%	Remifentanil group: remifentanil 0.83 ug/kg/min; small dose fentanyl group: fentanyl bolus, small dose, at 12 ug/kg; moderate dose fentanyl group: fentanyl bolus, moderate dose, at 24 ug/kg	All of remifentanil, small dose fentanyl and moderate dose fentanyl groups: propofol 100%	NR	Duration of mechanical ventilation, hospital-LOS, costs
Winterhalter et al. [33]	*European Journal of Anaesthesiology*	2008	42 mechanically ventilated postsurgical adult patients; mean age 63	ICU	100%	Remifentanil group: remifentanil 0.25 ug/kg/min; fentanyl group: fentanyl bolus 4 ug/kg every 30 min	Both of remifentanil and fentanyl groups: propofol 100%	VAS < 4	Duration of mechanical ventilation, hospital-LOS, time to extubation after cessation of sedation
Bedirli et al. [34]	*Journal of Anesthesia*	2007	50 mechanically ventilated postsurgical patients; mean age 61	ICU	100%	Remifentanil group: remifentanil 1ug/kg/min; fentanyl group: fentanyl 5 ug/kg/h	None	NR	Hospital-LOS;ICU-LOS
Chinachoti et al. [35]	*Medical Association of Thailand*	2002	152 mechanically ventilated patients with normal renal function or mild renal impairment; mean age 59; mean SAPS II 26	ICU	NR	Remifentanil group: 9–60 ug/kg/h; morphine group:0.045–0.3 mg/kg/h	Both of remifentanil and morphine groups: midazolam	SAS 4	Time to extubation after cessation of sedation, duration of mechanical ventilation

*APACHE* Acute Physiology and Chronic Health Evaluation, *BPS* Behavioural Pain Scale, *ICU* intensive care unit, *ICU-LOS* intensive care unit length of stay, *NR* not recorded, *SAPS* Simplified Acute Physiology Score, *SAS* Sedation Agitation Scale, *SOPA* Survey of Pain Attitudes, *VAS* Visual Analog Scale

Discordant opinions between the two reviewers were discussed until consensus was reached. If consensus could not be reached, a consulting group including two experts (XXM and DB) resolved the disagreements.

### Outcomes and statistical analysis

The primary outcome was duration of mechanical ventilation. The primary outcome was analysed in five subgroups: analgesia only; analgesia and sedation; and comparisons of remifentanil with fentanyl, morphine, and/or sufentanil. The secondary outcomes included (1) time to extubation after cessation of sedation; (2) ICU-LOS; (3) hospital-LOS; (4) costs; (5) mortality; and (6) agitation. Costs were measured in dollars, and other currencies converted into dollars according to the 2016 exchange rate. The costs were measured as overall costs, such as ICU or hospital costs. Maximum costs were preferentially used if a study reported more than one cost measure.

The pooled effects were analysed using weighted mean differences (WMDs) and 95% confidence intervals (CIs). The presence of statistically significant heterogeneity across trials was quantitatively assessed using the I^2^ statistic. Inverse variance random-effects models were applied for the data analysis. Publication bias was evaluated using funnel plots when at least ten studies were included in this meta-analysis. A *p* value less than 0.05 was considered statistically significant. All statistical analyses were performed using Review Manager Version 5.3.

## Results

### Study selection

**Fig. 1:**
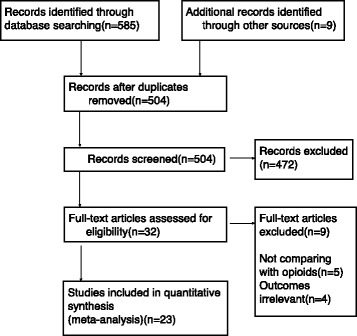
Flow diagram of the process for identification of the included studies

Overall, 585 potentially relevant articles were identified using the search strategy. After screening the titles/abstracts of the studies, 49 articles remained and were obtained in full-text form. Twenty-six studies failed to meet the previously described inclusion criteria; therefore, 23 studies were included in this meta-analysis. Of the included studies, 19 were published in English, two were published in French, one was published in Chinese, and one was published in Thai. No relevant unpublished studies were identified. Figure 1 presents the study selection process.

### Study characteristics and quality

A total of 1905 critically ill adult patients were included in the studies subjected to meta-analysis. The sample sizes of the 23 included studies ranged from 20 to 205 participants. Twenty-two studies compared remifentanil with another one or two opioids, and one study compared remifentanil/propofol with fentanyl/midazolam. Table 1 summarizes the basic characteristics of the included studies.

The results of the study quality assessment showed that five of the 23 RCTs were of high quality (Modified Jadad score 4–7), and the other 18 RCTs were of suboptimal quality (Modified Jadad score 0–4); these studies commonly lacked details regarding concealment of allocation and withdrawals and dropouts. Table 2 summarizes the quality of the included RCTs.

**Table 2:** Quality assessment of included RCTs

Study ID	Randomization	Concealment of allocation	Double blinding	Withdraws and dropouts	Modified Jadad score	Quality assessment
Al et al. [11]	2	0	0	0	2	Low
Rozendaal et al. [16]	2	0	0	0	2	Low
Spies et al. [13]	2	2	2	1	7	High
Liu et al. [20]	2	0	0	0	2	Low
Khanykin et al. [36]	2	0	0	1	3	Low
Bhavsar et al. [21]	2	0	0	0	2	Low
Engoren et al. [12]	2	0	0	0	2	Low
Muellejans et al. [22]	1	0	0	1	2	Low
Muellejans et al. [23]	1	0	1	1	3	Low
Karabinis et al. [24]	1	0	0	0	1	Low
Baillard et al. [25]	2	0	1	0	3	Low
Dahaba et al. [1]	2	2	2	1	7	High
Breen et al. [26]	1	0	0	0	1	Low
Belhadj Amor et al. [27]	2	0	2	0	4	Low
Carrer et al. [28]	1	0	0	0	1	Low
Gerlach et al. [29]	2	2	0	0	4	High
Guggenherger et al. [30]	2	0	0	1	3	Low
Knapik et al. [31]	1	0	0	0	1	Low
Maddali et al. [32]	2	0	0	1	3	Low
Myles et al. [19]	2	0	2	1	5	High
Winterhalter et al. [33]	2	2	2	0	6	High
Bedirli et al. [34]	2	0	0	0	2	Low
Chinachoti et al. [35]	2	0	1	0	3	Low

### Outcomes

**Fig. 2:**
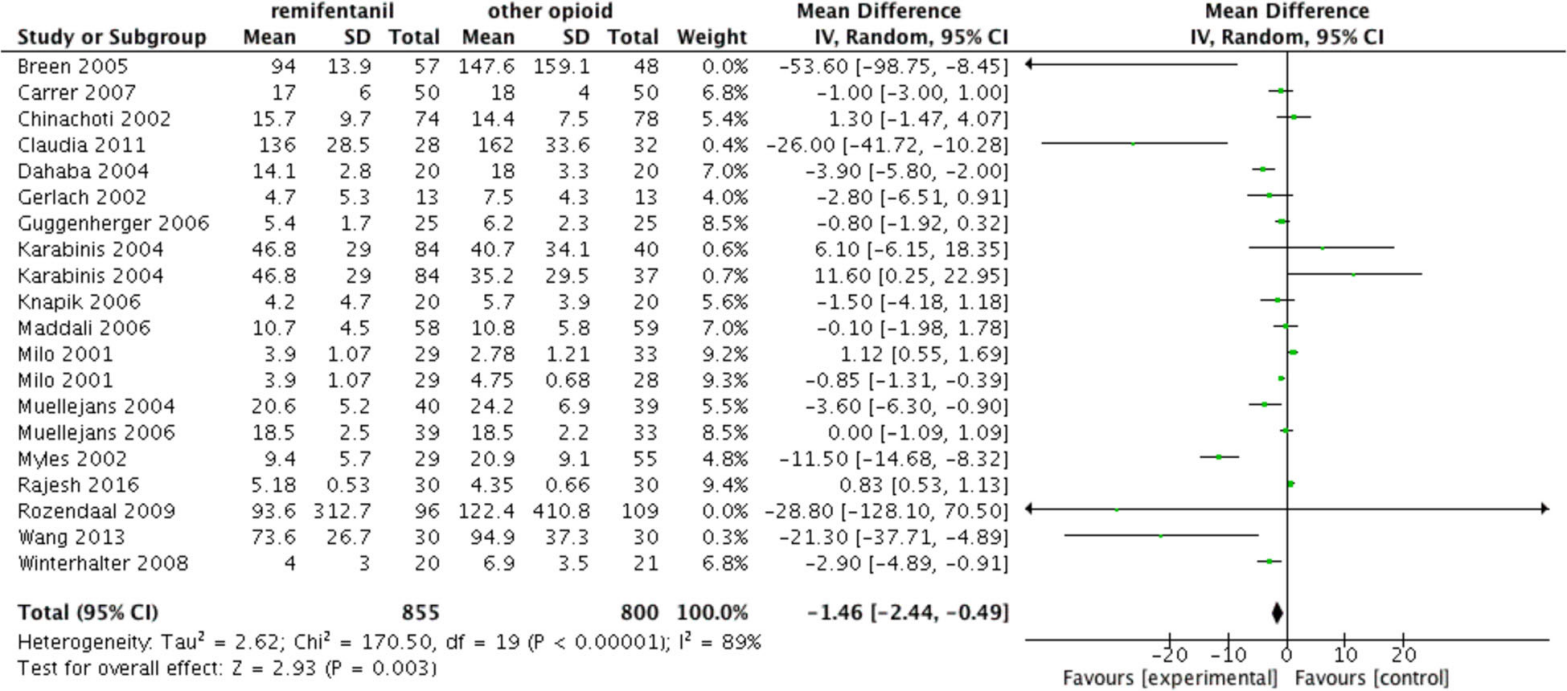
Primary outcome. Remifentanil was associated with a reduction in duration of mechanical ventilation

The primary outcome, duration of mechanical ventilation, was reported in 18 RCTs. Remifentanil was associated with a reduction in the duration of mechanical ventilation (mean difference -1.46; 95% CI -2.44 to -0.49; I^2^ 89%. Fig. 2a).

**Fig. 3:**
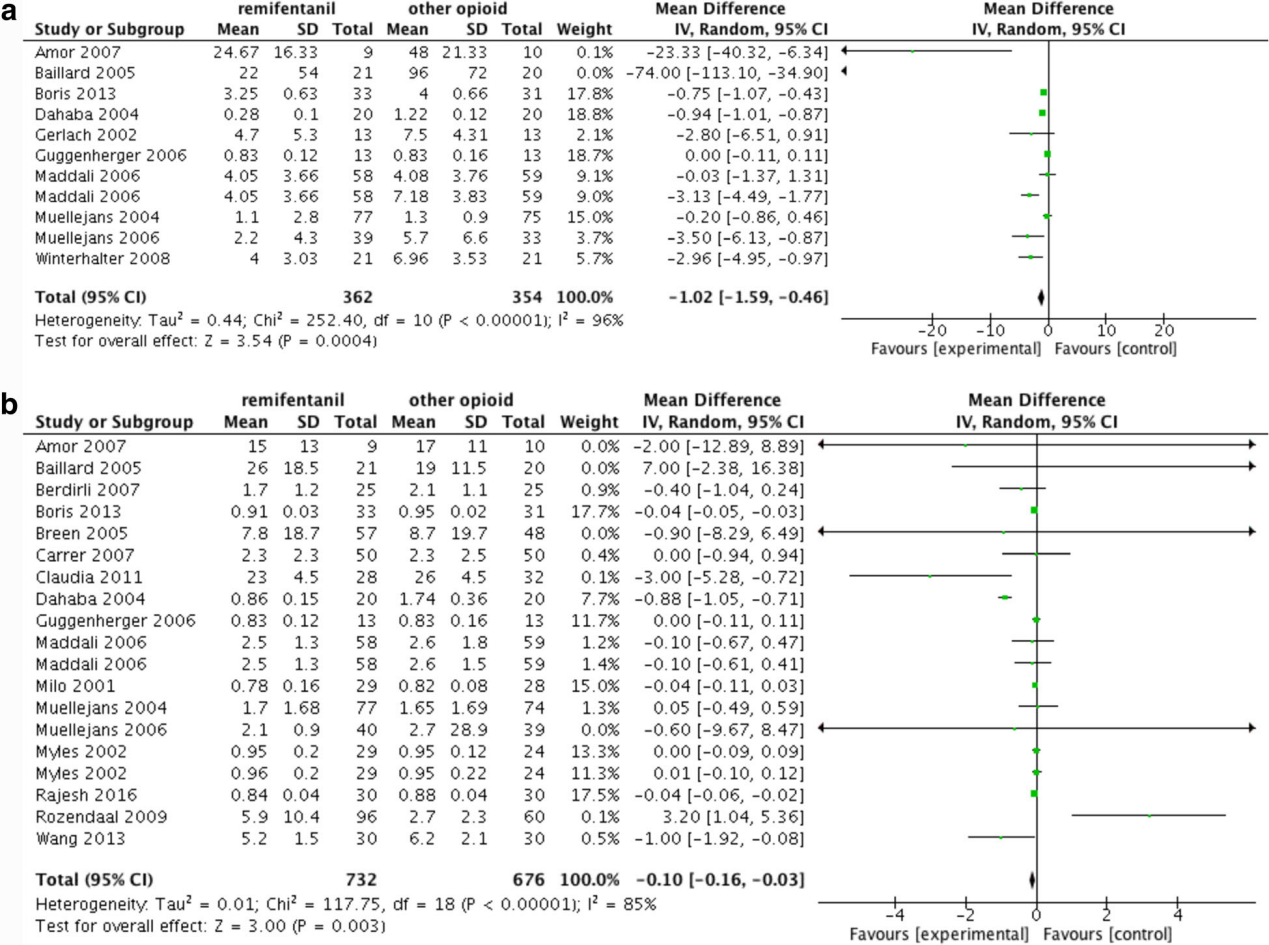
Secondary outcomes. Remifentanil was associated with a reduction in time to extubation after cessation (**a**) and ICU-LOS (**b**)

As for the secondary outcomes, remifentanil was associated with reductions in time to extubation after sedation cessation (mean difference -1.02; 95% CI -1.59 to -0.46; I^2^ 96%. Fig. 3a) and ICU-LOS (mean difference -0.10; 95% CI -0.16 to -0.03; I^2^ 85%. Fig. 3b). No significant differences in hospital-LOS (mean difference -0.05; 95% CI -0.25 to 0.15; I^2^ 88%. Additional file 2: Figure S2a), costs (mean difference -709.71; 95% CI -1590.98 to 171.55; I^2^ 88%. Additional file 2: Figure S2b), mortality (mean difference -0.64; 95% CI -1.33 to 0.06; I^2^ 87%. Additional file 2: Figure S2c) and agitation (mean difference -0.71; 95% CI -1.80 to 0.37; I^2^ 93%. Additional file 2: Figure S2d) were identified. Table 3 summarizes the data for and analyses of outcome measures.

**Table 3:** Data and analyses of outcome measures

Outcome	Studies	Participants	Effect estimate	Heterogeneity (I^2^)	Measure
Duration of mechanical ventilation (Fig. 1)	18	1655	-1.46 [-2.44]	89%	Hour
Subgroup of analgesia only (Additional file 3: Figure S1a)	4	291	0.14 [-0.79]	91%	Hour
Subgroup of analgesia and sedation (Additional file 3: Figure S1b)	14	1364	-2.99 [-5.09]	84%	Hour
Subgroup of remifentanil comparing with fentanyl (Additional file 3: Figure S1c)	8	624	-3.85 [-7.39]	93%	Hour
Subgroup of remifentanil comparing with morphine (Additional file 3: Figure S1d)	4	416	-0.98 [-3.81]	74%	Hour
Subgroup of remifentanil comparing with remifentanil (Additional file 3: Figure S1e)	5	233	-0.58 [-1.78]	91%	Hour
Time to extubation after cessation of sedation (Fig. 2)	10	716	-0.68 [-0.74]	96%	Hour
ICU-LOS (Fig. 2)	17	1408	-0.04 [-0.05]	85%	Day
Hospital-LOS (Additional file 2: Figure S2a)	9	507	-0.05 [-0.25]	88%	Day
Costs (Additional file 2: Figure S2b)	4	437	943.54 [-1122.69]	90%	Dollar
Mortality (Additional file 2: Fig. S2c)	10	260	-0.64 [-1.33]	87%	Person
Agitation (Additional file 2: Fig S2d)	3	184	-0.71 [-1.80]	93%	Person
Delirium (Additional file 2: Figure S2e)	4	323	1.01 [0.63]	0	Person

As for the subgroup analyses, remifentanil was associated with a reduction in mechanical ventilation duration in the subgroup of studies in which both analgesia and sedation were administered (mean difference -2.99; 95% CI -5.09 to -0.89; I^2^ 84%. Additional file 3: Figure S1a) and the subgroup of studies in which remifentanil and fentanyl were compared (mean difference -3.85; 95% CI -7.39 to -0.31; I^2^ 93%. Additional file 3: Figure S1c). No significant differences were identified in the subgroup of studies in which only analgesia was administered (mean difference 0.14; 95% CI -0.79 to 1.07; I^2^ 91%. Additional file 3: Figure S1a), the subgroup of studies in which remifentanil and morphine were compared (mean difference -0.98; 95% CI -3.81 to 1.85; I^2^ 74%. Additional file 3: Figure S1d), and the subgroup of studies in which remifentanil and sufentanil were compared (mean difference -0.58; 95% CI -1.78 to 0.62; I^2^ 91%. Additional file 3: Figure S1e).

### Assessment of publication biases

**Fig. 4:**
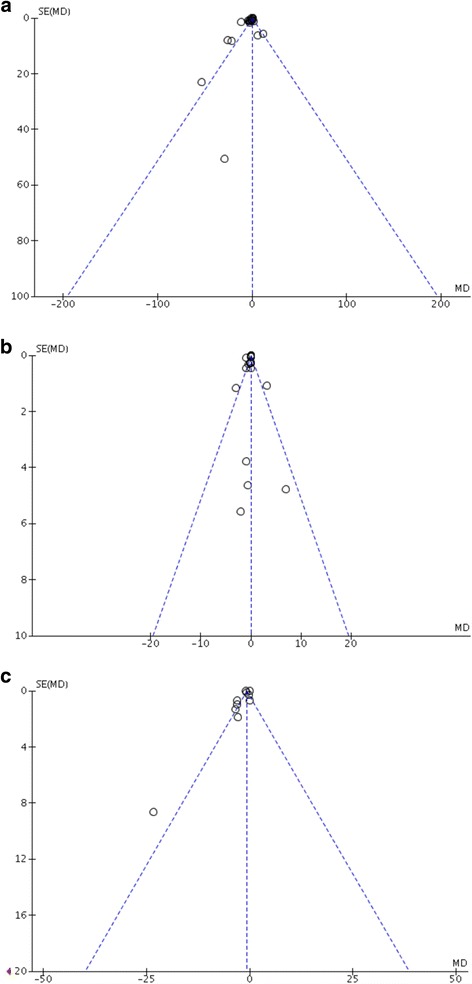
Funnel plots. Funnel plots were generally asymmetrical. The *hollow dots* and *dotted line* indicate individual studies and 95% confidence intervals, respectively. **a** Funnel plot of duration of mechanical ventilation. **b** Funnel plot of ICU-LOS. **c** Funnel plot of time to extubation after cessation of sedation

Biases in the publication of the three outcome measures (duration of mechanical ventilation, time to extubation after cessation of sedation, and ICU-LOS) were evaluated using funnel plots. The funnel plots depicted in Fig. 4 were generally asymmetrical, which indicated the presence of publication bias. The points representing the evaluated studies in the three funnel plots were concentrated at the top showed that the studies had high precision and large sample sizes.

## Discussion

The results of this meta-analysis suggested that the use of remifentanil was associated with a reduction in the duration of mechanical ventilation when compared with other opioids, findings that were supported by the results of subgroup of studies in which analgesia and sedation were administered and subgroup of studies in which remifentanil was compared with fentanyl; however, the subgroups of studies in which analgesia alone was administered and studies in which remifentanil was compared with morphine or sufentanil indicated the presence of no differences. Remifentanil was associated with reductions in time to extubation after sedation cessation and ICU-LOS but not reductions in hospital-LOS or costs.

The finding that remifentanil was found to reduce the duration of mechanical ventilation, time to extubation after sedation cessation, and ICU-LOS may be highly related to the unique pharmacokinetics and pharmacodynamics of remifentanil, namely, its rapid onset and offset, context-sensitive half-life that is hardly affected by the duration of infusion, and elimination that is independent of liver or renal function [14, 15]. In the subgroups, the results of the studies comparing remifentanil with fentanyl showed a maximal mean difference of 3.85 hours. As a selective μ-opioid receptor agonist [16, 17], remifentanil was similar to fentanyl in potency but different in pharmacokinetics. This result may indicate that remifentanil may be used as a substitute for fentanyl in some circumstances, especially in cases in which patients have developed kidney injuries or in which intermittent interruption of sedation and analgesia is needed to assess altered mental status. However, these results should be interpreted with caution for a few reasons. First, the mean differences between remifentanil and other opioids were only 1.5 hours for mechanical ventilation duration, 1 hour for time to extubation after sedation cessation, and 0.1 day for IC-LOS, which suggested that only miniscule differences may be identified in clinical practice. However, the potential benefits of remifentanil might be more apparent when used in patients with significant organ failure [6, 14], a hypothesis that more studies should explore. Moreover, neurologic assessment is essential for neurosurgical and neurotrauma patients; thus, the association between remifentanil and rapid and predictable awakening may be more meaningful in these patients, even though the difference between remifentanil and other opioids were less than 1 hour [5]. Second, high levels of heterogeneity were identified for all of the outcomes. There were remarkable differences across the included RCTs in terms of type of disease, analgesic agents and sedation protocols. We analysed the outcomes in subgroups classified by the use of different control groups and sedation protocols to reduce clinical heterogeneity; however, the statistical homogeneity was still obvious (I^2^ > 70%, considered as high heterogeneity). In addition, we selected a random-effects model rather than fixed-effects model to address the observed heterogeneity. Third, the funnel plots suggested the presence of publication bias in the three evaluated outcomes, which may be because the pharmaceutical companies that manufactured remifentanil funded some of the included studies.

Remifentanil was not associated with a reduction of hospital-LOS, costs, mortality, of agitation, and no differences were observed in the subgroups of studies in which only analgesia was administered and subgroup of studies in which remifentanil was compared with morphine or sufentanil, which may be because remifentanil and other opioids are similar in most regards; however, these results should be interpreted with caution for a few reasons. First, these outcome measures were assessed in small samples, and high homogeneity was observed. The mortality rate was low and not statistically powered to assess certain clinical outcomes [18]. Second, the combination of sedatives and analgesics made the estimation of the effect of opioids more difficult. Moreover, the sedation protocols and agents differed from study to study. Third, hospital-LOS and costs may be mainly affected by the severity of diseases rather than the selection of analgesia agents. In addition, anaesthetic costs accounted for only a small fraction of the overall costs. Costs were highly variable, with 95% CI ranging from -1590.98 to 171.55 dollars. Only four RCTs included assessments of costs [11, 12, 19, 20]. The types of diseases that patients were affected by in the four studies varied considerably, and the mean Acute Physiology and Chronic Health Evaluation II (APACHE II) score varied from 20.1 to 46, suggesting the presence of large variations in cost. Further cost-effectiveness studies are needed to explore the association between analgesic agents and cost.

The strengths of our meta-analysis include the structured search strategy, retrieval of all identified studies and large sample size. Taking the measurement of mechanical ventilation duration as an example, we included 18 RCTs in the comparisons, while the previous meta-analysis only included four RCTs [9] and we believe that our results might be more convincing than the results of the previous meta-analysis due to the inclusion of a larger sample size of patients.

There are limitations to our meta-analysis. First, the choice of hypnotic differed widely from one study to another, and the analysis of the effects of hypnotic choice was, thus, more difficult to perform. Second, both clinical and statistical heterogeneities were high. In addition, most of the included RCTs (78%) were of suboptimal quality. Third, we were unable to exclude publication bias, and negative studies may be missing, potentially resulting in overestimation of the effect sizes.

Taken together, remifentanil seems to be associated with reductions in the duration of mechanical ventilation, time to extubation after sedation cessation, and ICU-LOS. No significant differences were identified between remifentanil and other opioids in terms of hospital-LOS, costs, mortality or agitation.

## Conclusions

Remifentanil seems to be associated with reductions in the duration of mechanical ventilation and time to extubation after sedation cessation. Additional studies are needed to further evaluate the efficacy and safety of remifentanil and the association between the use of remifentanil and cost in critically ill patients, especially patients undergoing long-term mechanical ventilation.

## Additional files

Additional file 1: Appendix 1: PubMed search strategy. (PDF 18 kb)

Additional file 2: Figure S2. Secondary outcomes. There was no significant difference in hospital-LOS (a), costs (b), mortality (c) and agitation (d) in comparison with remifentanil and other opioids. (PDF 92 kb)

Additional file 3: Figure S1. Subgroup analyses. Remifentanil was associated with a reduction in duration of mechanical ventilation in subgroups of analgesia and sedation (b) and remifentanil comparing with fentanyl (c). There was no significant difference in subgroups of analgesia only (a), morphine (d), and sufentanil (e). (PDF 112 kb)

## Abbreviations

APACHE II: Acute Physiology and Chronic Health Evaluation II
CI: Confidence interval
hospital-LOS: Length of stay in hospital
ICU: Intensive care unit
ICU-LOS: Length of stay in ICU
RA: Remifentanil acid
RCT: Randomized controlled trial
SAPS II: Simplified Acute Physiology Score II
WMD: Weighted mean difference
