# Supplementary material for: Could remifentanil reduce duration of mechanical ventilation in comparison with other opioids for mechanically ventilated patients? A systematic review and meta-analysis
Source: Crit Care. 2017 Aug 3;21:206. doi: 10.1186/s13054-017-1789-8 (PMC5543734; doi:10.1186/s13054-017-1789-8)
Supplement: Supplementary file 1 — Appendix 1: PubMed search strategy. (PDF 18 kb) [file 13054_2017_1789_MOESM1_ESM.pdf]

PubMed Search strategy

1#(((((((((((critical care[Title/Abstract]) OR ICU[Title/Abstract]) OR intensive care[Title/Abstract]) OR critical ill[Title/Abstract]) OR critical illness[Title/Abstract]) OR critically ill[Title/Abstract])) OR ((("Intensive Care Units"[Mesh]) OR "Critical Care"[Mesh]) OR "Critical Illness"[Mesh]))))) OR mechanical ventilation[Title/Abstract]) OR mechanically ventilated[Title/Abstract]) OR ventilation[Title/Abstract]

2#remifentanil[Title/Abstract]

3# 1# and 2#
