# Supplementary material for: Could remifentanil reduce duration of mechanical ventilation in comparison with other opioids for mechanically ventilated patients? A systematic review and meta-analysis
Source: Crit Care. 2017 Aug 3;21:206. doi: 10.1186/s13054-017-1789-8 (PMC5543734; doi:10.1186/s13054-017-1789-8)
Supplement: Supplementary file 2 — Secondary outcomes. There was no significant difference in hospital-LOS (a), costs (b), mortality (c) and agitation (d) in comparison with remifentanil and other opioids. (PDF 92 kb) [file 13054_2017_1789_MOESM2_ESM.pdf]

Study or Subgroup	remifentanil Mean	SD	Total	other opioid Mean	SD	Total	Weight	Mean Difference IV, Fixed, 95% CI
Baillard 2005	8.8	2.9	25	7.4	2.6	25	1.7%	1.40 [−0.13, 2.93]
Boris 2013	5	0.7	33	5	0.5	31	43.8%	0.00 [−0.30, 0.30]
Claudia 2011	33	4.5	28	39	4.8	32	0.7%	−6.00 [−8.35, −3.65]
Guggenherger 2006	10	2	25	12	1	25	5.0%	−2.00 [−2.88, −1.12]
Knapik 2006	11.1	9.4	20	9.1	2.7	20	0.2%	2.00 [−2.29, 6.29]
Milo 2001	5	0.7	29	5	0.8	28	25.2%	0.00 [−0.39, 0.39]
Myles 2002	4.9	2.1	29	5.9	2	55	4.5%	−1.00 [−1.93, −0.07]
Rajesh 2016	7.9	1	30	7.1	0.9	30	16.6%	0.80 [0.32, 1.28]
Winterhalter 2008	9	2	21	10	2.3	21	2.3%	−1.00 [−2.30, 0.30]
**Total (95% CI)**			**240**			**267**	**100.0%**	**−0.05 [−0.25, 0.15]**

Heterogeneity: $Chi^2 = 66.09$, df = 8 ($P < 0.00001$); $I^2 = 88\%$
Test for overall effect: Z = 0.49 (P = 0.62)

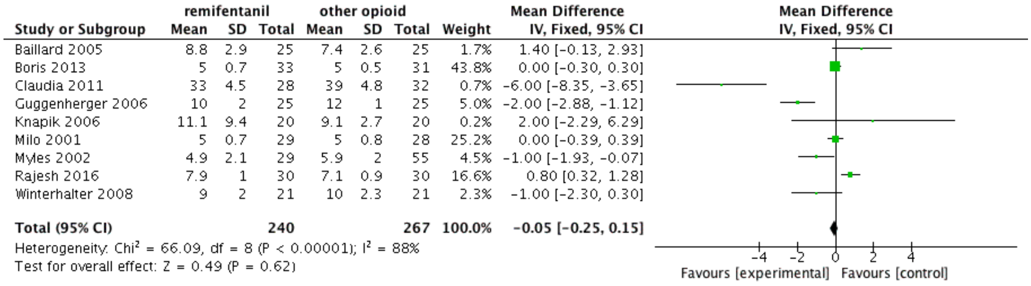

a. hospital-LOS

Study or Subgroup	remifentanil Mean	SD	Total	other opioid Mean	SD	Total	Weight	Mean Difference IV, Random, 95% CI
Maiwenn 2010	16,315	1,561	96	17,871	1,359	109	21.5%	−1556.00 [−1959.23, −1152.77]
Milo 2001	6,286	934	29	5,943	1,087	28	20.8%	343.00 [−183.94, 869.94]
Milo 2001	6,286	834	29	7,841	1,154	33	21.0%	−1555.00 [−2052.15, −1057.85]
Myles 2002	14,456	2,377	27	15,734	4,232	23	10.7%	−1278.00 [−3226.12, 670.12]
Myles 2002	14,456	1,377	27	14,634	3,374	23	13.8%	−178.00 [−1651.47, 1295.47]
Wang 2013	8,714	3,292	30	8,383	3,422	30	12.2%	331.00 [−1368.16, 2030.16]
**Total (95% CI)**			**238**			**246**	**100.0%**	**−709.71 [−1590.98, 171.55]**

Heterogeneity: $Tau^2 = 899551.32$; $Chi^2 = 40.67$, df = 5 ($P < 0.00001$); $I^2 = 88\%$
Test for overall effect: Z = 1.58 (P = 0.11)

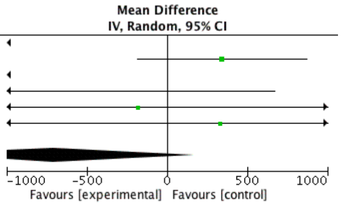

b. costs

Study or Subgroup	Remifentanil Mean	SD	Total	Control Mean	SD	Total	Weight	Mean Difference IV, Random, 95% CI
Bedirli 2007	8.8	2.9	25	7.4	2.6	25	8.8%	1.40 [−0.13, 2.93]
Boris 2013	5	0.7	33	5	0.5	31	14.7%	0.00 [−0.30, 0.30]
Claudia 2011	33	4.5	28	39	4.8	32	5.6%	−6.00 [−8.35, −3.65]
Guggenberger 2006	10	2	25	12	1	25	12.2%	−2.00 [−2.88, −1.12]
Knapik 2006	11.1	9.4	20	9.1	2.7	20	2.3%	2.00 [−2.29, 6.29]
Milo 2001	5	0.7	29	5	0.8	28	14.5%	0.00 [−0.39, 0.39]
Myles 2002	4.9	2.1	29	5.9	2	55	11.9%	−1.00 [−1.93, −0.07]
Rajesh 2016	7.9	1	30	7.1	0.9	30	14.1%	0.80 [0.32, 1.28]
Winterhalter 2008	9	2	21	10	2.3	21	9.9%	−1.00 [−2.30, 0.30]
Wong 2010	11	3	20	13	4	20	6.1%	−2.00 [−4.19, 0.19]
**Total (95% CI)**			**260**			**287**	**100.0%**	**−0.64 [−1.33, 0.06]**

Heterogeneity: $Tau^2 = 0.84$; $Chi^2 = 69.11$, df = 9 ($P < 0.00001$); $I^2 = 87\%$
Test for overall effect: Z = 1.79 (P = 0.07)

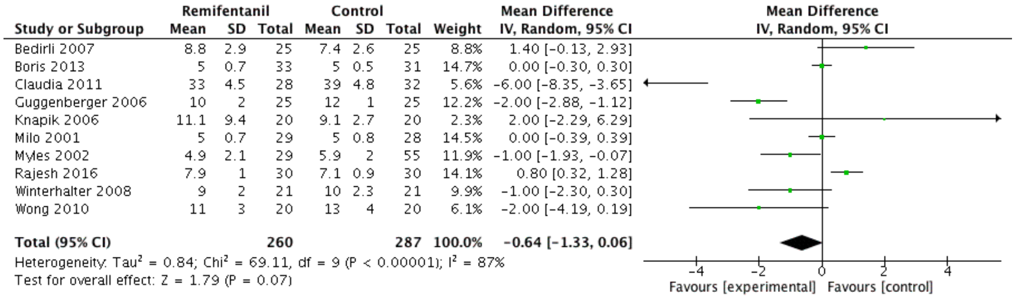

c.mortality

Study or Subgroup	Remifentanil Mean	SD	Total	Control Mean	SD	Total	Weight	Mean Difference IV, Random, 95% CI
Maiwenn 2010	16.315	1.561	96	17.871	1.359	109	28.1%	−1.56 [−1.96, −1.15]
Milo 2001	6.286	0.834	29	7.841	1.154	33	27.5%	−1.56 [−2.05, −1.06]
Milo 2001	6.286	0.834	29	5.943	1.087	28	27.5%	0.34 [−0.16, 0.85]
Wang 2013	8.714	3.292	30	8.383	3.422	30	17.0%	0.33 [−1.37, 2.03]
**Total (95% CI)**			**184**			**200**	**100.0%**	**−0.71 [−1.80, 0.37]**

Heterogeneity: $Tau^2 = 1.04$; $Chi^2 = 41.70$, df = 3 ($P < 0.00001$); $I^2 = 93\%$
Test for overall effect: Z = 1.29 (P = 0.20)

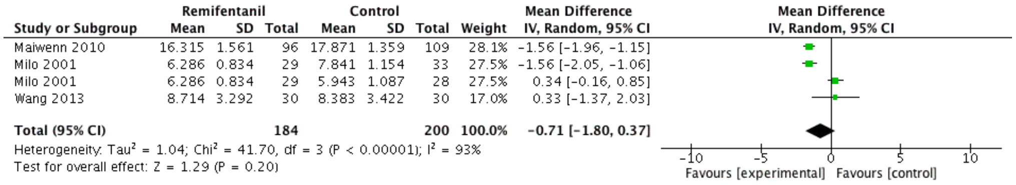

d.agitation

Fig. S2 Secondary outcomes. There was no significant difference in hospital-LOS (a), costs (b), mortality (c) and agitation (d) in comparison with remifentanil and other opioids.
