# Supplementary material for: Could remifentanil reduce duration of mechanical ventilation in comparison with other opioids for mechanically ventilated patients? A systematic review and meta-analysis
Source: Crit Care. 2017 Aug 3;21:206. doi: 10.1186/s13054-017-1789-8 (PMC5543734; doi:10.1186/s13054-017-1789-8)
Supplement: Supplementary file 3 — Subgroup analyses. Remifentanil was associated with a reduction in duration of mechanical ventilation in subgroups of analgesia and sedation (b) and remifentanil comparing with fentanyl (c). There was no significant difference in subgroups of analgesia only (a), morphine (d), and sufentanil (e). (PDF 112 kb) [file 13054_2017_1789_MOESM3_ESM.pdf]

Study or Subgroup	remifentanil Mean	SD	Total	other opioid Mean	SD	Total	Weight	Mean Difference IV, Random, 95% CI
Knapik 2006	4.2	4.7	20	5.7	3.9	20	8.2%	-1.50 [-4.18, 1.18]
Milo 2001	3.9	1.07	29	2.78	1.21	33	23.5%	1.12 [0.55, 1.69]
Milo 2001	3.9	1.07	29	4.75	0.68	28	24.2%	-0.85 [-1.31, -0.39]
Muellejans 2006	18.5	2.5	39	18.5	2.2	33	19.1%	0.00 [-1.09, 1.09]
Rajesh 2016	5.18	0.53	30	4.35	0.66	30	25.1%	0.83 [0.53, 1.13]
**Total (95% CI)**			**147**			**144**	**100.0%**	**0.14 [-0.79, 1.07]**

Heterogeneity: Tau² = 0.88; Chi² = 44.24, df = 4 (P < 0.00001); I² = 91%
Test for overall effect: Z = 0.30 (P = 0.76)

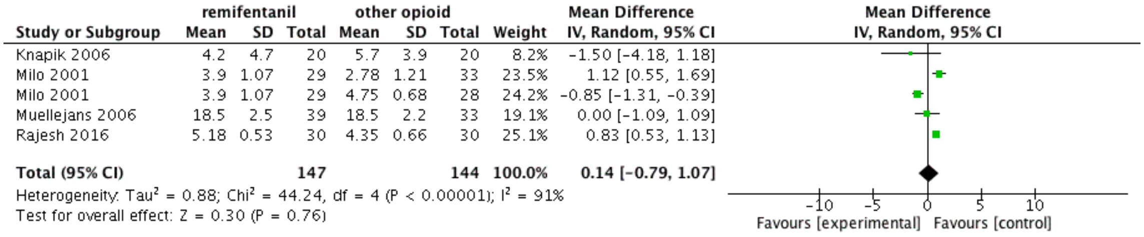

a.subgroup of analgesia only

Study or Subgroup	remifentanil Mean	SD	Total	other opioid Mean	SD	Total	Weight	Mean Difference IV, Random, 95% CI
Breen 2005	94	13.9	57	147.6	159.1	48	0.2%	-53.60 [-98.75, -8.45]
Carrer 2007	17	6	50	18	4	50	10.6%	-1.00 [-3.00, 1.00]
Chinachoti 2002	15.7	9.7	74	14.4	7.5	78	9.7%	1.30 [-1.47, 4.07]
Claudia 2011	136	28.5	28	162	22.6	32	2.1%	-26.00 [-39.14, -12.86]
Dahaba 2011	14.1	2.8	20	18	3.3	20	10.7%	-3.90 [-5.80, -2.00]
Gerlach 2002	4.7	5.3	13	7.5	4.3	13	8.6%	-2.80 [-6.51, 0.91]
Guggenherger 2006	5.4	1.7	25	6.2	2.3	25	11.3%	-0.80 [-1.92, 0.32]
Karabinis 2004	46.8	29	84	40.7	34.1	40	2.3%	6.10 [-6.15, 18.35]
Karabinis 2004	46.8	29	84	35.2	29.5	37	2.6%	11.60 [0.25, 22.95]
Maddali 2006	10.7	4.5	58	10.8	5.8	59	10.7%	-0.10 [-1.98, 1.78]
Muellejans 2004	20.6	5.2	40	24.2	6.9	39	9.8%	-3.60 [-6.30, -0.90]
Myles 2002	9.4	5.7	29	20.9	9.1	55	9.2%	-11.50 [-14.68, -8.32]
Rozendaal 2009	93.6	312.7	96	122.4	410.8	109	0.0%	-28.80 [-128.10, 70.50]
Wang 2013	73.6	26.7	30	94.9	37.3	30	1.4%	-21.30 [-37.71, -4.89]
Winterhalter 2008	4	3	20	6.9	3.5	21	10.6%	-2.90 [-4.89, -0.91]
**Total (95% CI)**			**708**			**656**	**100.0%**	**-2.99 [-5.09, -0.89]**

Heterogeneity: Tau² = 9.81; Chi² = 85.88, df = 14 (P < 0.00001); I² = 84%
Test for overall effect: Z = 2.79 (P = 0.005)

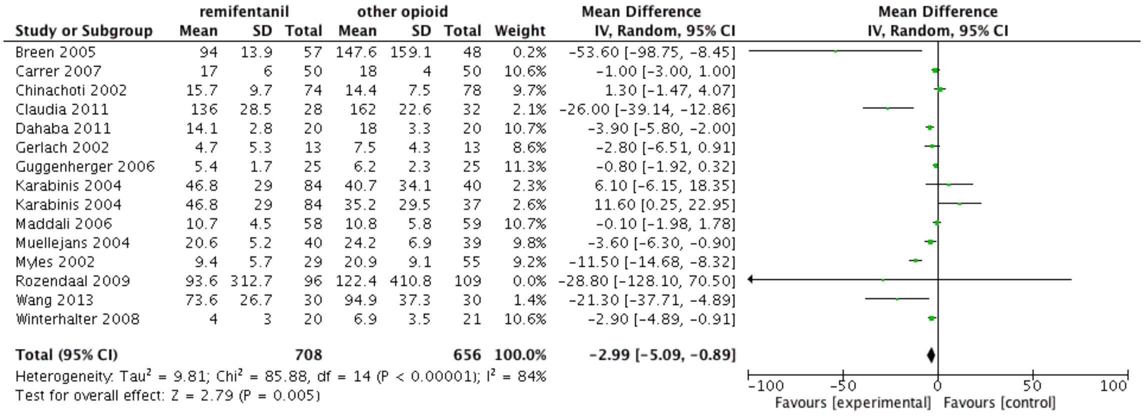

b.subgroup of analgesia and sedation

Study or Subgroup	remifentanil Mean	SD	Total	fentanyl Mean	SD	Total	Weight	Mean Difference IV, Random, 95% CI
Winterhalter 2008	4	3	20	6.9	3.5	21	17.4%	-2.90 [-4.89, -0.91]
Wang 2013	73.6	26.7	30	94.9	37.3	30	3.7%	-21.30 [-37.71, -4.89]
Myles 2002	9.4	5.7	29	20.9	9.1	55	16.0%	-11.50 [-14.68, -8.32]
Muellejans 2004	20.6	5.2	40	24.2	6.9	39	16.6%	-3.60 [-6.30, -0.90]
Milo 2001	3.9	1.07	29	2.78	1.21	33	18.3%	1.12 [0.55, 1.69]
Maddali 2006	10.7	4.5	58	10.8	5.8	59	17.5%	-0.10 [-1.98, 1.78]
Karabinis 2004	46.8	29	84	35.2	29.5	37	6.4%	11.60 [0.25, 22.95]
Claudia 2011	136	28.5	28	162	33.6	32	4.0%	-26.00 [-41.72, -10.28]
**Total (95% CI)**			**318**			**306**	**100.0%**	**-3.85 [-7.39, -0.31]**

Heterogeneity: Tau² = 17.73; Chi² = 100.33, df = 7 (P < 0.00001); I² = 93%
Test for overall effect: Z = 2.13 (P = 0.03)

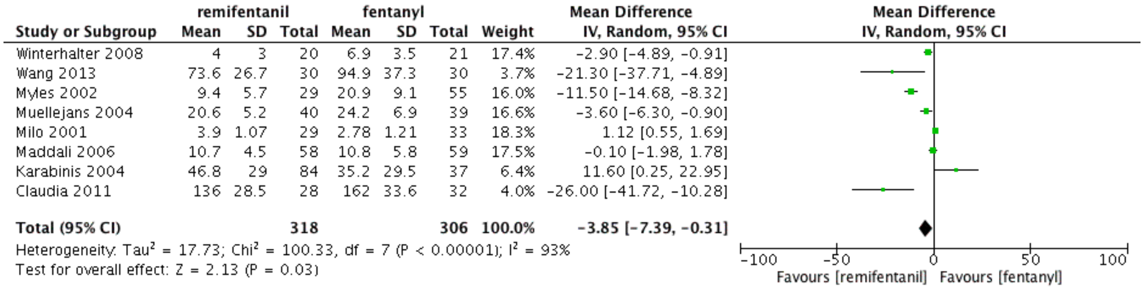

c.subgroup of remifentanil comparing with fentanyl

Study or Subgroup	remifentanil Mean	SD	Total	morphine Mean	SD	Total	Weight	Mean Difference IV, Random, 95% CI
Carrer 2007	17	6	50	18	4	50	33.0%	-1.00 [-3.00, 1.00]
Chinachoti 2002	15.7	9.7	74	14.4	7.5	78	28.7%	1.30 [-1.47, 4.07]
Dahaba 2004	14.1	2.8	20	18	3.3	20	33.6%	-3.90 [-5.80, -2.00]
Karabinis 2004	46.8	29	84	40.7	34.1	40	4.7%	6.10 [-6.15, 18.35]
**Total (95% CI)**			**228**			**188**	**100.0%**	**-0.98 [-3.81, 1.85]**

Heterogeneity: Tau² = 5.28; Chi² = 11.71, df = 3 (P = 0.008); I² = 74%
Test for overall effect: Z = 0.68 (P = 0.50)

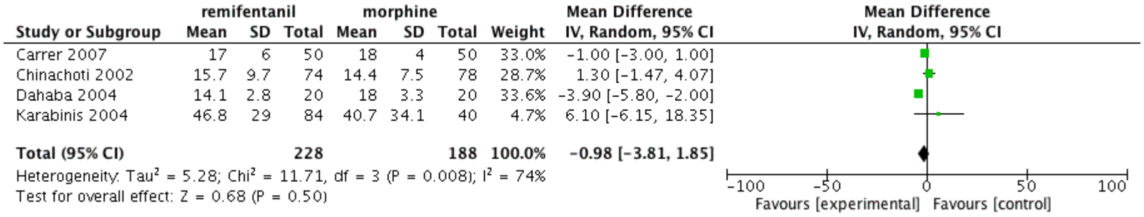

d.subgroup of remifentanil comparing with morphine

Study or Subgroup	remifentanil Mean	SD	Total	sufentanil Mean	SD	Total	Weight	Mean Difference IV, Random, 95% CI
Gerlach 2002	4.7	5.3	13	7.5	4.3	13	7.7%	-2.80 [-6.51, 0.91]
Guggenherger 2006	5.4	1.7	25	6.2	2.3	25	23.4%	-0.80 [-1.92, 0.32]
Knapik 2006	4.2	4.7	20	5.7	3.9	20	11.9%	-1.50 [-4.18, 1.18]
Milo 2001	3.9	1.07	29	4.75	0.68	28	28.1%	-0.85 [-1.31, -0.39]
Rajesh 2016	5.18	0.53	30	4.35	0.66	30	28.8%	0.83 [0.53, 1.13]
**Total (95% CI)**			**117**			**116**	**100.0%**	**-0.58 [-1.78, 0.62]**

Heterogeneity: Tau² = 1.27; Chi² = 43.30, df = 4 (P < 0.00001); I² = 91%
Test for overall effect: Z = 0.95 (P = 0.34)

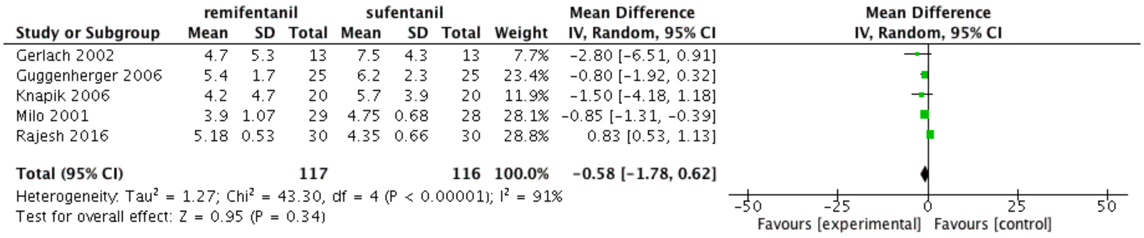

e.subgroup of remifentanil comparing with sufentanil

Fig. S1 Subgroup analyses. Remifentanil was associated with a reduction in duration of mechanical ventilation in subgroups of analgesia and sedation (b) and remifentanil comparing with fentanyl (c). There was no significant difference in subgroups of analgesia only (a), morphine (d), and sufentanil (e).
